# Baron Larrey on Prolapsus Uteri

**Published:** 1831-01-01

**Authors:** 


### On Prolapsus Uteri. By Baron Larrey.

The venerable Baron has not confined his attention solely to diseases and wounds of soldiers. His talents have been often directed to the alleviation of those maladies which afflict civil life.

Prolapsus uteri, to a greater or less extent, is not uncommon among women who have a shallow pelvis, and who have borne many children. One of the most frequent causes of this complaint is the dangerous traction of the funis, after delivery of the fœtus, by ignorant midwives. The complaint is more common in France than in England on this account. The prolapsus exists in various degrees. When the uterus comes to the external orifice of the vagina—when the subject is of a relaxed fibre, and an advanced age—there is no other resource but to return the organ to its place, and to endeavour to support it there by means of a pessary. But if the subject be young, and the organ does not fall lower than the inferior orifice of the pelvis, great hopes may be entertained of a cure—and of the prevention of scirrhus of the uterus, which, he thinks, is often the result of the prolapsus. This complaint, he conceives, is owing to two causes—one is, an asthenic engorgement of the parietes of the organ—the other, an elongation or relaxation of the ligaments, produced in the manner above alluded to, or by large utero-fœtations. The remedial measures embrace cupping the loins, the groins, and other places as near as possible to the seat of the engorgement. When the local depletion is effected, Baron Larrey applies the Moxa, two at a time, to the parts above-mentioned. He remarks that we need not fear to reiterate the applications from time to time, assisting the measure by the horizontal posture, elevation of the pelvis, and flexion of the lower extremities—to which may be added cold and astringent applications and injections, laxative lavements, and mild nutritious diet. By two, three, or four months of this treatment, he has succeeded in completely curing prolapsus uteri. We shall introduce one or two cases in illustration.

*Case* 1. Mad. de V——, aged 27 years, consulted the Baron for a prolapsus uteri of the third degree, which had succeeded a second accouchement, where great and unnecessary traction of the placenta had been employed. She could not bear the pessary. The means above-mentioned were put in use, and in 84 days the cure was complete. The horizontal posture was, of course, maintained during that time. Baron Larrey employed similar measures in a great number of other cases, and with the same result.

*Case* 2. Madame de C——, aged 27 years, tall, and of exquisite sensibility, after a laborious fourth accouchement, became affected with a prolapsus uteri, where the organ appeared visible at the os externum. She consulted an accoucheur, who ordered her to wear a pessary. The introduction of this instrument was difficult and painful—and its remaining there was excessively annoying, preventing the passage of urine and stools, except with great pain. After six weeks' perseverance in this plan, she consulted Baron Larrey, who proposed the removal of the pessary, and the adoption of the means already described. She complied with his proposal, and in six months, the cure was so complete that this young lady could take long and fatiguing journeys on foot, without any inconvenience.

